# 883. The Impact of Geographic Distance on Pediatric Burn Severity and Clinical Outcomes

**DOI:** 10.1093/jbcr/irag033.369

**Published:** 2026-04-06

**Authors:** Sandra Bojic, Jakob Weirathmueller, Claudia C Malic

**Affiliations:** University of Ottawa, Ottawa, ON; University of Ottawa, Ottawa, ON; University of Ottawa, Ottawa, ON

## Abstract

**Introduction:**

Health disparities are often associated with geographic barriers to specialized care. This study aimed to examine how distance from a specialized pediatric burn center relates to burn severity and clinical outcomes. The center serves a vast catchment area, including remote populations with limited access to specialized burn care.

**Methods:**

A retrospective review of 2013 pediatric burn cases over a 9-year period (2016-2024) was conducted. Patient postal codes were geocoded to calculate straight-line distance to the center. Associations with outcomes were analyzed using linear regression and ANOVA, while Geographic Information Systems (GIS) identified regional clusters.

**Results:**

Burn cases concentrated around the burn center, with another hotspot in remote northern regions. Greater distance was significantly associated with increased burn severity, with average burn depth (*p*=.0438) and TBSA increasing from 2.7% to 5.2% (*p*=.0180) from the <25 km to the >200 km cohort. Patients residing 100-200 km and > 200 km had odds ratios of 3.54 and 4.39 for requiring skin grafting compared to those <25 km. Conversely, pre-hospital first aid significantly reduced likelihood of skin grafting (OR 0.59). Patients 100-200 km and > 200 km had significantly lower rates of first aid (43.1% and 45.2%) compared to <25 km (59.7%; *p*=.003).

**Conclusions:**

Farther distance correlated with higher surgery rates. Children >200 km away were four times more likely to require skin grafts, presenting with larger and deeper burns. It is likely that smaller burns were managed locally, and only patients meeting referral criteria were transferred. Children farther from a specialized center are typically referred when their burns are more severe. These findings highlight the utility of GIS for identifying at-risk areas and underscore the need for prevention campaigns targeting areas with limited access to specialized care.

**Applicability of Research to Practice:**

These findings support implementation of distance-based interventions including expanded telehealth consultations for remote regions, targeted first aid education programs in areas >100 km from the burn center, and region-specific prevention campaigns in identified geographic hotspots. Early first aid intervention shows significant protective effects and should be prioritized in remote area education initiatives.

**Funding for the study:**

N/A.

